# Functionally Graded Piezoelectric Medium Exposed to a Movable Heat Flow Based on a Heat Equation with a Memory-Dependent Derivative

**DOI:** 10.3390/ma13183953

**Published:** 2020-09-07

**Authors:** Ahmed E. Abouelregal, Hijaz Ahmad, Shao-Wen Yao

**Affiliations:** 1 Department of Mathematics, College of Science and Arts, Jouf University, Al-Qurayyat 77413, Saudi Arabia; ahabogal@mans.edu.eg; 2 Department of Mathematics, Faculty of Science, Mansoura University, Mansoura 35516, Egypt; 3 Department of Basic Sciences, University of Engineering and Technology, Peshawar 25000, Pakistan; hijaz555@gmail.com; 4 School of Mathematics and Information Science, Henan Polytechnic University, Jiaozuo 454000, China

**Keywords:** thermo-piezoelectric, memory-dependent, functionally graded, kernel function, electric potential

## Abstract

The current work deals with the study of a thermo-piezoelectric modified model in the context of generalized heat conduction with a memory-dependent derivative. The investigations of the limited-length piezoelectric functionally graded (FGPM) rod have been considered based on the presented model. It is assumed that the specific heat and density are constant for simplicity while the other physical properties of the FGPM rod are assumed to vary exponentially through the length. The FGPM rod is subject to a moving heat source along the axial direction and is fixed to zero voltage at both ends. Using the Laplace transform, the governing partial differential equations have been converted to the space-domain, and then solved analytically to obtain the distributions of the field quantities. Numerical computations are shown graphically to verify the effect of memory presence, graded material properties, time-delay, Kernel function, and the thermo-piezoelectric response on the physical fields.

## 1. Introduction

The classical coupled Fourier heat conduction model is no longer valid and can only predict the unlimited speed of heat propagation. To eliminate inconsistencies and defects in the classical theory, several extensions to the Fourier heat conduction law have been introduced. As an extended thermoelasticity theory, Lord and Shulman [1] presented the generalized theory of thermoelasticity with one relaxation time by proposing another law of heat conduction to supplant the classical Fourier’ law. The equation of heat of this theory is of the wave-sort which guarantees limited paces of the proliferation of heat and elastic waves.

Functionally graded (FG) rods have many valuable applications, as they are mainly utilized as structural members in numerous industrial applications like spacecraft, petrochemical structures, and nuclear equipment. One of the most important physical properties of functionally graded material (FGM) is that they work under very high thermal conditions, which in turn may lead to vibratory motion, particularly when exposed to an unexpected change in thermal conditions [2]. In addition to the above, mechanical behavior and control of material vibration can be improved further by presenting piezoelectric layers onto their internal or/and external surfaces [3]. These novel nonhomogeneous materials have excellent thermo-mechanical properties and have broad applications to vital structures, for example, atomic reactors, pressure vessels, chemicals plants, aviation, and channels, and so on. The problems of a functionally graded elastic half-space are considered by many researchers [4,5,6,7,8,9].

The propagation of waves in thermo-piezoelectric sensors and actuators was the focus of Paul Langevin and other investigators to research on the invention of the hydrophone during World War I, after the Curie brothers’ discovery of piezoelectricity in 1880. Piezoelectric materials are broadly utilized as a part of brilliant structures and materials as actuators and sensors, etc. The piezo-thermoelastic theory was initially proposed by Mindlin [10]. The physical laws explored by Nowacki [11,12] for the known thermopiezoelectric material, while the Mindlin’s theory of piezo-thermoelastic has been generalized by Chandrasekharaiah [13] to represent the limited speed of spread of thermal unsettling influences.

Since piezoelectric materials can be used in many applications, especially where the environment is thermogenic where temperature variation is often one of the most significant criteria in analyzing their behavior, many investigators have attempted to take in this in governing equations for the study of thermopiezoelectricity [14,15,16,17,18]. Also, in this regard, Abouelregal [19] applied the fractional-order thermoelasticity model for a thermo-piezoelectric semi-infinite medium concerned to a ramp-type heating and temperature-dependent properties. Shaw and Mukhopadhyay [20] considered the thermoelastic interaction in a functionally graded medium. Mallik and Kanoria [21] considered the thermoelastic interaction in functionally graded an unbounded solid because of varying heat source. Zenkour and Abouelregal [22,23,24] considered the effect of two temperatures and thermoelastic vibration induced by a pulse heating on a functionally graded nanobeam. Abouelregal and Zenkour [25] investigated the effect of thermoelastic phase lags and ramp-type heating on functionally graded microbeams. Othmani et al. [26] conducted a theoretical study on the scattering curves of lamb waves in GaAs-FGPMAlAs piezoelectrical-semiconductor plates. In the practical piezoelectric plate consisting of GaAs-AlAs materials, Othmani et al. [27] introduced computational simulation of the propagation of Lamb waves using the Legendre polynomial method.

In the last two decades, a great interest has been devoted to fractional calculus due to its applications in various fields of biology, rheology, viscoelasticity, biophysics, electrical chemistry, bioengineering and electrical engineering, signal processing and images, physics and mechanics. Currently, to improve the classical Fourier law, some research has been done on heat conduction by fractional calculus, see [28,29,30,31,32,33,34].

By incorporating a memory-dependent derivative (MDD) into the Fourier law of heat conduction, a novel heat conduction equation of a hyperbolic type is presented by Wang and Li [35]. This new model has studied a generalization of the theory of the heat conduction with memory. In this context, some thought-provoking thermoelastic investigations based on the memory-dependent differential equation can be found in [36,37,38,39,40,41,42].

Taking benefit of these advantages of the coupling between the mechanical and electric fields in piezoelectric materials, piezoelectric is wide as smart structures like power transformers, actuators and sensors. The optimal design of these smart structures needs the advancement of theoretical tools to explore a variety of problems related to piezoelectric materials under different loading conditions.

In the current work, a problem thermo-piezoelectricity is considered in the light of a novel memory-dependent heat conduction model. The thermoelastic and piezoelectric coupled equations for a finite piezoelectric rod are derived and resolved by employing the Laplace transform. The solutions of the displacement, temperature, stress and electric potential have been attained analytically. Some comparisons in order to assess the results are offered graphically, which revealed the effects of the memory dependent, nonhomogeneity index and piezoelectric on all studied fields.

## 2. Fundamental and Governing Equations

A better definition than a fractional to reverse the effect of memory, a memory-dependent derivative is defined in an integral form of a common derivative with a kernel function over a sliding interval, which is more intuitive to comprehend the physical meanings. Furthermore, fractional theory has been applied in practically every area of science: viscoelasticity and rheology, electrical, electrochemical, biochemical, biophysical and bioengineering processing, signals and images, dynamics, dynamics, physics and control theory.

The Caputo derivative of fractional order α (n−1≤α≤n) of function f(t) defined as [43]

(1) $$D_{a}^{\alpha }f\left(t\right)=\frac{1}{\Gamma \left(n-\alpha \right)}\int_{a}^{t}\frac{D^{n}f\left(\tau \right)}{{\left(t-\tau \right)}^{\alpha -n+1}}d\tau ,$$

where $D^{n}$ is *nth* derivative operation, Γ(n−α) is the Gamma function, f(t) is a Lebesgue integrable function and t is the time.

The use of the memory-dependent derivative (MDD) in the heat conduction equation means that the heat transport equation is modified, and the current formula, which is more than fractional, must also be improved. Taking into account the disparity between these two models, the current model is special though fractional-order models include different images in the various authors. The new model is also more precisely described by integer-order differentials and integrals; this is more suitable for numerical than fractional calculations, and, finally, the kernel function and time delay of an MDD can be chosen arbitrarily.

Recently, Diethelm [40] reviewed the extension from the common integer-order differentiation and integration to the fractional derivative and developed the Caputo [43] derivative to be:

(2) $$D_{a_{0}}^{\left(\alpha \right)}f\left(x,t\right)=\int_{a_{0}}^{t}k\left(t-\xi \right)f^{\left(m\right)}\left(\xi \right)d\xi ,t\in \left[a_{0},b_{0}\right]$$

With $k\left(t-\xi \right)=\frac{{\left(t-\xi \right)}^{m-\alpha -1}}{\Gamma \left(m-\alpha \right)}$.

Where m is an integer, α satisfies m−1<α≤m, $f^{\left(m\right)}\left(\xi \right)$ shows the usual m-th order derivative, and k(t−ξ) is the kernel function which can be chosen freely with 0≤k(t−ξ)≤1.

In Wang and Li [27], a memory- dependent derivative (MDD) was introduced in the first order of the function f(t), defined in the form of a single integrated derivative with a slip interval kernel function [t−ω,t]:

(3) $$D_{\omega }f\left(t\right)=\frac{1}{\omega }\int_{t-\omega }^{t}k\left(t-\xi \right)f{}^{\prime }\left(\xi \right)d\xi$$

ω>0 being the time delay whereas k(t−ξ) is the kernel function which can be chosen freely with 0≤k(t−ξ)≤1. The kernel function k(t−ξ) is utilized to measure the degree of memory effect from past to the present in the delayed interval [t−ω,t]. The above modifications of the fractional-order derivatives are termed as MDD.

The classical theory of the heat conduction based on Fourier’s law is given by

(4) $$q_{i}=-K_{ij}\theta_{,j},\;\;\;\;i,j,k=1,2,3$$

where $K_{ij}$ are the coefficients of thermal conductivity, and $\theta =T-T_{0}$ is the dynamical temperature increment, where $T_{0}$ is the initial temperature. Based on a rate-type constitutive equation, unlike Fourier theory, the Maxwell–Cattaneo theory is given by

(5) $$q_{i}+\tau_{0}\frac{\partial q_{i}}{\partial t}=-K_{ij}\theta_{,j},\;\;\;\;i,j,k=1,2,3$$

In [35] Yu et al. inserted the memory dependent derivatives (MDD) into the Lord–Shulman (LS) generalized thermoelasticity theory in the rate of heat flux to show the memory dependence in the beneath way:

(6) $$\left(1+\tau_{0}\;D_{\omega }\right)q_{i}=-K_{ij}\theta_{,j},$$

where $D_{\omega }$ is the MDD of the first order of the heat flux may be defined by

(7) $$D_{\omega }^{\left(1\right)}q_{i}\left(x,t\right)=\frac{1}{\omega }\int_{t-\omega }^{t}k\left(t-\xi \right)q_{i}^{\prime }\left(x,\xi \right)d\xi$$

According to brothers Jacques and Pierre Curie [44], piezoelectric influence is the capability of certain crystalline materials to develop an electric charge proportional to mechanical stress. Hence, piezoelectricity is the contact between mechanical and electrical systems. The constitutive equations for piezoelectric media give the coupling between the electrical and mechanical parts of the piezoelectric system.

Therefore, the constitutive equations of stress, electric displacement, strain–displacement relation, entropy for a linear piezo-thermoelastic material are [13,19]

(8) $$\sigma_{ij}=C_{ijkl}\varepsilon_{kl}-e_{ijk}E_{k}-\beta_{ij}\theta ,\;$$

(9) $$\rho S=\frac{\rho C_{E}}{T_{0}}\theta +\beta_{ij}\varepsilon_{ij}+p_{i}E_{i},\;\;\;\;\;$$

(10) $$D_{i}=e_{ijk}\varepsilon_{kl}+\in_{ij}E_{j}+p_{i}\theta ,\;$$

(11) $$2\varepsilon_{ij}=u_{i,j}+u_{j,i}.$$

In these equations, $\varepsilon_{ij}$ are the strain tensors, $\sigma_{ij}$ are the stress tensors, $u_{i}$ are the components of the displacement vector, S is the entropy, $D_{i}$ are the components of the electric flux density, $q_{i}$ is heat flux, $C_{ijkl}$ are elastic coefficients, $e_{ijk}$ are the piezoelectric coefficients, $\beta_{ij}$ are the thermal moduli, $C_{E}$ is the specific heat per unit mass at constant strain, ρ is the material density of the medium, $p_{i}$ is the pyroelectric coefficients. Also, $\in_{ij}$ are dielectric coefficients, $\tau_{0}$ is the thermal relaxation time, the coefficient $K_{ij}$ is for thermal conductivity, $E_{i}$ represent the electric field and $\theta =T-T_{0}$ is the dynamical temperature increment, where $T_{0}$ is the initial temperature. The equations of motion for a piezoelectric medium without the body forces and energy equation [10,45]:

(12) $$\sigma_{ji,j}=\rho \;{\ddot{u}}_{i},$$

(13) $$\rho T_{0}\frac{\partial S}{\partial t}-\rho Q+q_{i,i}=0,$$

The magnetic and electric fields in a medium are defined by Maxwell’s equation (Coulomb equation), which relate the fields to the microscopic average properties of the material. The beneath Coulomb equation is also required for the description of a piezoelectric medium without free charge and electric current

(14) $$D_{i,i}=0,$$

When the quasistatic approximation is presented in [46], the electric field E is derivable from a scalar electric potential φ as

(15) $$E_{i}=-\varphi_{,i}$$

With the use of Equations (6), (9) and (13), equation of heat conduction in the framework of generalized piezo-thermoelasticity theory with one relaxation time:

(16) $${\left(K_{ij}\theta_{,j}\right)}_{,i}=\left(1+\tau_{0}D_{\omega }\right)\left(\rho C_{E}\frac{\partial \theta }{\partial t}+\beta_{ij}\;T_{0}\frac{\partial u_{m,m}}{\partial t}-T_{0}p_{k}{\dot{E}}_{k}\right)$$

In the absence of piezoelectric effects ($e_{ijk},\in_{ij},p_{i}\to 0$), the constitutive Equations (1)–(5) and the field equations reduce to the generalized thermoelasticity [1]. Also, avoiding thermal relaxation time (i.e., $\tau_{0}\to 0$), we get the equations of the coupled thermo-piezoelectricity theory. Furthermore, if $e_{ijk},\in_{ij},p_{i},\beta_{ij}\to 0$ and $\tau_{0}\to 0$ classical Fourier heat conduction can also be obtained.

In this paper, the kernel function k(t−ξ) can be chosen freely as [47]

(17) $$k\left(t-\xi \right):=1-\frac{2b}{\omega }\left(t-\xi \right)+\frac{a^{2}{\left(t-\xi \right)}^{2}}{\omega^{2}}=\{\begin{array}{c} 1,\;\;\;\;\;\;\;\;\;\;\;\;\;\;\;\;\;\;\;\;\;\;\;\;if\;a=b=0\\ 1-\frac{\left(t-\xi \right)}{\omega },\;\;\;\;\;\;\;\;\;\;\;\;\;\;\;if\;a=0,\;b=\frac{1}{2}\\ 1-\left(t-\xi \right),\;\;\;\;\;\;\;\;\;\;\;\;if\;a=0,\;b=\omega /2\;\\ {\left(1-\frac{\left(t-\xi \right)}{\omega }\right)}^{2},\;\;\;\;\;\;\;\;if\;a=1,\;b=1 \end{array}$$

where a,b are constant.

## 3. Statement of the Problem

In this section, we have considered the vibrations in a functionally graded thermo-piezoelectric finite rod of length L rely on the Lord–Shulman model. The medium is thought to be initially at a uniform temperature $T_{0}$ and covering the region z≥0. The surface of the medium is subjected to a heat source that appears at (z=0) moving along the medium with a constant speed υ. For the current one-dimensional problem, we assume that stress, electric displacement, displacement, electric field, strain and heat flux components vanish with the exception of the components in the z direction and depending just on the length z and time t. At that point the strain components $\varepsilon_{ij}$ and electric field E get to be

(18) $$\varepsilon_{zz}=\frac{\partial w\left(z,t\right)}{\partial z},\;\;\;\;\;\;\;\;\;\;\;E_{z}=-\frac{\partial \varphi }{\partial z}.$$

Equations (12) and (13), and the constitutive relations have given in (8)–(11) are reduced to

(19) $$\frac{\partial \sigma_{zz}}{\partial z}=\rho \frac{\partial^{2}w}{\partial t^{2}},$$

(20) $$\rho T_{0}\frac{\partial S}{\partial t}-\rho Q+\frac{\partial q_{z}}{\partial z}=0,$$

(21) $$\sigma_{zz}=C\frac{\partial w}{\partial z}+e\frac{\partial \varphi }{\partial z}-\beta \theta ,$$

(22) $$\rho S=\frac{\rho C_{E}}{T_{0}}\theta +\beta \frac{\partial w}{\partial z}-p\frac{\partial \varphi }{\partial z},\;$$

(23) $$D_{z}=e\frac{\partial w}{\partial z}-\in \frac{\partial \varphi }{\partial z}+p\theta ,\;$$

(24) $$q_{z}+D_{\omega }\frac{\partial q_{z}}{\partial t}=-K\frac{\partial \theta }{\partial z}$$

The piezo-thermoelastic heat conduction equation MDD (16) can be expressed as

(25) $$\frac{\partial }{\partial z}\left(K\frac{\partial \theta }{\partial z}\right)=\left(1+\tau_{0}D_{\omega }\right)\left(\rho C_{E}\frac{\partial \theta }{\partial t}+\beta \;T_{0}\frac{\partial w}{\partial t\partial z}+T_{0}p\frac{\partial \varphi }{\partial t\partial z}-\rho Q\right)$$

From Equation (14), we get the electrical displacement as

(26) $$D\left(z\right)=D_{0}=\mathrm{constant}.$$

Replacing Equation (26) into Equation (24), the potential gradient equation:

(27) $$\frac{\partial \varphi }{\partial z}=\frac{e}{\in }\frac{\partial w}{\partial z}-\frac{D_{0}}{\in }+\frac{p}{\in }\theta ,$$

Introducing Equation (27) into Equations (21) and (25), the thermal stress $\sigma_{zz}$ and the heat equation can be rewritten as

(28) $$\sigma_{zz}=\left(C+\frac{e^{2}}{\in }\right)\frac{\partial w}{\partial z}-\frac{eD_{0}}{\in }+\left(\frac{ep}{\in }-\beta \right)\theta ,$$

(29) $$K\frac{\partial^{2}\theta }{\partial z^{2}}+\frac{\partial K}{\partial z}\frac{\partial \theta }{\partial z}=\left(1+\tau_{0}D_{\omega }\right)\left(\left(\rho C_{E}+\frac{T_{0}p^{2}}{\in }\right)\frac{\partial \theta }{\partial t}+T_{0}\left(\beta +\frac{p^{2}}{\in }\right)\;\frac{\partial w}{\partial t\partial z}-\rho Q\right)$$

The properties of composite materials like FGMs, change continuously and gradually along a definite direction in the domain of material. Because of the effects of functionally graded and nonhomogeneous solid, the physical properties of the materials are no longer fixed but have become dependent on space. Since the rod’s materials are thought to be graded along with the z-directions, the material properties aside from thermal relaxation time $\tau_{0}$ and specific heat $C_{E}$ have been assumed to be described with the exponential form as [9,48]

(30) $$\xi \left(z\right)=\xi_{0}e^{nz},$$

where ξ(z) is a generalized material property, $\xi_{0}$ is a constant represents the matching value of the property at z=0 and n is a nonhomogeneity arbitrary index. Substitution of the relation (30) into the governing Equations (28)–(30), we have

(31) $$\frac{\partial \varphi }{\partial z}=\frac{e_{0}}{\in_{0}}\frac{\partial w}{\partial z}+\frac{p_{0}}{\in_{0}}\theta -\frac{D_{0}}{\in_{0}}e^{-nz},$$

(32) $$\sigma_{zz}=e^{nz}\left[\left(C_{0}+\frac{e_{0}^{2}}{\in_{0}}\right)\frac{\partial w}{\partial z}+\left(\frac{e_{0}p_{0}}{\in_{0}}-\beta_{0}\right)\theta \right]-\frac{e_{0}D_{0}}{\in_{0}},$$

(33) $$K_{0}\left(\frac{\partial^{2}\theta }{\partial z^{2}}+n\frac{\partial \theta }{\partial z}\right)=\left(1+\tau_{0}D_{\omega }\right)\left(\left(\rho_{0}C_{E}+\frac{T_{0}p_{0}^{2}}{\in_{0}}\right)\frac{\partial \theta }{\partial t}+T_{0}\left(\beta_{0}+\frac{p_{0}^{2}}{\in_{0}}\right)\;\frac{\partial w}{\partial t\partial z}-\rho_{0}Q\right)$$

The moving heat supply along the *z*-axis may be defined in the nondimensional form as [46]:

(34) $$Q\left(z,t\right)=Q_{0}\delta \left(z-\upsilon t\right),$$

where υ and $Q_{0}$ denote the velocity and intensity of the heat source respectively, whereas δ(.) is Dirac delta function. To investigate the problem, we define the following nondimensional parameters:

(35) $$\begin{array}{c} \left(z^{\prime },w^{\prime }\right)=v\eta \left(z,w\right),\;\;\;\left(t^{\prime },{\tau^{\prime }}_{0}\right)=v^{2}\eta \left(t,\tau_{0}\right),\;\;\;\theta^{\prime }=\frac{\theta }{T_{0}},\;\;\;{\sigma^{\prime }}_{zz}=\frac{\sigma_{zz}}{C_{0}},\\ {D^{\prime }}_{z}=\frac{D_{z}}{C_{0}},\varphi^{\prime }=\frac{v\eta \in_{0}}{e_{0}}\varphi ,\;\;\;\;Q^{\prime }=\frac{Q}{v^{2}\eta^{2}K_{0}T_{0}},\;\;\;\;\;v^{2}=\frac{C_{0}}{\rho_{0}},\;\;\;\;\;\eta =\frac{\rho_{0}C_{E}}{K_{0}},\;l=Lv\eta \end{array}$$

After omitting dashes, equations, the governing equations in nondimensional form can be expressed as

(36) $$\frac{\partial \varphi }{\partial z}=\frac{\partial w}{\partial z}+h_{12}\theta -h_{13}e^{-Nz},$$

(37) $$\sigma_{zz}=e^{Nz}\left[h_{21}\frac{\partial w}{\partial z}-h_{22}\theta \right]-h_{23},$$

(38) $$\left(\frac{\partial^{2}\theta }{\partial z^{2}}+N\frac{\partial \theta }{\partial z}\right)=\left(1+\tau_{0}D_{\omega }\right)\left(h_{31}\frac{\partial \theta }{\partial t}+h_{32}\;\frac{\partial w}{\partial t\partial z}-Q\right)$$

where

(39) $$\begin{array}{c} \;h_{12}=\frac{T_{0}p_{0}}{e_{0}},\;\;\;\;h_{13}=\frac{C_{0}D_{0}}{e_{0}},\;\;N=\frac{n}{v\eta },\;\;h_{21}=\frac{C_{0}\in_{0}+e_{0}^{2}}{C_{0}\in_{0}},\;h_{23}=\frac{e_{0}D_{0}}{\in_{0}},\;\\ \;h_{22}=\frac{T_{0}\left(\in_{0}\beta_{0}-e_{0}p_{0}\right)}{C_{0}\in_{0}},\;\;\;\;h_{31}=\frac{K_{0}\eta \in_{0}+T_{0}p_{0}^{2}}{K_{0}\eta \in_{0}},\;\;h_{32}=\frac{\beta_{0}}{K_{0}\eta }+\frac{p_{0}^{2}}{K_{0}\eta \in_{0}}. \end{array}$$

Substituting Equations (36) and (37) into Equation (19), the equation of motion of piezo-thermoelasticity is:

(40) $$h_{21}\left(\frac{\partial^{2}w}{\partial z^{2}}+N\frac{\partial w}{\partial z}\right)-h_{22}\left(\frac{\partial \theta }{\partial z}+N\theta \right)=\rho_{0}v^{3}\eta \frac{\partial^{2}w}{\partial t^{2}}$$

## 4. Initial and Boundary Conditions

The initial-boundary conditions need to be considered for the solution of the present problem. The initial conditions of the proposed problem are:

(41) $${w\left(z,t\right)|}_{t=0}={\frac{\partial w\left(z,t\right)}{\partial t}|}_{t=0}=0,\;\;{\theta \left(z,t\right)|}_{t=0}={\frac{\partial \theta \left(z,t\right)}{\partial t}|}_{t=0}=0.$$

We suppose that the medium is thermally insulated at z=0, L and fixed with zero voltage at the surface z=0. So the following boundary conditions hold:

(42) φ(0,t)=0 

(43) $$w\left(0,t\right)=w\left(L,t\right)=\frac{\partial \theta \left(0,t\right)}{\partial z}=\frac{\partial \theta \left(L,t\right)}{\partial z}=0$$

## 5. Solution in the Transformed Domain

Taking the Laplace transform of Equations (36)–(40), having the initial conditions (41), the transformed equations can be obtained as:

(44) $$\frac{d\bar{\varphi }}{dz}=\frac{d\bar{w}}{dz}+h_{12}\bar{\theta }-h_{13}e^{-Nz}/s,$$

(45) $${\bar{\sigma }}_{zz}=e^{Nz}\left[h_{21}\frac{d\bar{w}}{dz}-h_{22}\bar{\theta }\right]-h_{23}/s,$$

(46) $$\frac{d^{2}\bar{\theta }}{dz^{2}}+N\frac{d\bar{\theta }}{dz}=s\left(1+\frac{\tau_{0}}{\omega }\bar{G}\left(s,\omega \right)\right)\;\left(h_{31}\bar{\theta }+h_{32}\;\frac{dw}{dz}-\frac{Q_{0}}{\upsilon }e^{-\left(s/\upsilon \right)z}\right)$$

(47) $$h_{21}\left(\frac{d^{2}\bar{w}}{dz^{2}}+N\frac{d\bar{w}}{dz}\right)-h_{22}\left(\frac{d\bar{\theta }}{dz}+N\bar{\theta }\right)=\rho_{0}v^{3}\eta s^{2}\bar{w}$$

Equations (46) and (47) can be rewritten as

(48) $$\left(\frac{d^{2}}{dz^{2}}+N\frac{d}{dz}-h_{41}\right)\;\bar{\theta }=h_{42}\frac{dw}{dz}-h_{43}e^{-\left(s/\upsilon \right)z}$$

(49) $$h_{22}\left(\frac{d}{dz}+N\right)\;\bar{\theta }=h_{21}\left(\frac{d^{2}}{dz^{2}}+N\frac{d}{dz}-h_{44}\right)\bar{w}$$

where

(50) $$\begin{array}{c} h_{41}=s\left(1+\frac{\tau_{0}}{\omega }\bar{G}\left(s,\omega \right)\right)h_{31},\;\;h_{42}=s\left(1+\frac{\tau_{0}}{\omega }\bar{G}\left(s,\omega \right)\right)h_{32},\\ h_{43}=s\left(1+\frac{\tau_{0}}{\omega }\bar{G}\left(s,\omega \right)\right)Q_{0}/\upsilon ,\;\;h_{44}=\rho_{0}v^{3}\eta s^{2}/h_{21}. \end{array}$$

Moreover, one can show that for any function f(t) with first-order MDD, the Laplace transform is given by

(51) $$\begin{array}{c} L\left[\omega D_{\omega }f\left(t\right)\right]=L\left[\int_{t-\omega }^{t}k\left(t-\xi \right)f^{\prime }\left(\xi \right)d\xi \right]=L\left[f\left(t\right)\right]\;G\left(s,\omega \right);\\ G\left(s,\omega \right)=\left(1-e^{-s\omega }\right)\;\left[1-\frac{2b}{\omega \;s}+\frac{2a^{2}}{\omega^{2}s^{2}}\right]-\left[a^{2}-2b^{2}+\frac{2a^{2}}{\omega s}\right]e^{-s\omega } \end{array}$$

If kernel function in the MDD is constant i.e., when k(t−ξ)=1 then,

(52) $$G\left(s,\omega \right)=\left(1-e^{-s\omega }\right).\;$$

Eliminating $\bar{\theta }$ from Equations (48) and (49), one gets the following differential equation

(53) $$\left(\frac{d^{4}}{dz^{4}}+a_{3}\frac{d^{3}}{dz^{3}}-a_{2}\frac{d^{2}}{dz^{2}}-a_{1}\frac{d}{dz}+a_{0}\right)\;\bar{w}=m_{0}e^{-\left(s/\upsilon \right)z},$$

where

(54) $$\begin{array}{c} a_{3}=2N,\;\;\;\;\;\;\;a_{2}=h_{41}+N^{2}+h_{44}+h_{42}h_{22}/h_{21},\;\;a_{0}=h_{44}h_{41}\\ a_{1}=Nh_{41}+Nh_{44}+Nh_{42}h_{22}/h_{21},m_{0}=h_{43}h_{22}\left(s/v-N\right)/h_{21}\;. \end{array}$$

The characteristic equation of Equation (53) is given by

(55) $$k^{4}+a_{3}k^{3}-a_{2}k^{2}-a_{1}k+a_{0}=0$$

and $k_{i}$, i=1,2,3,4 are given by

(56) $$\begin{array}{c} k_{1}=-\frac{a_{3}}{4}-\frac{y_{6}}{2}-\frac{y_{8}}{2},\;\;\;\;\;k_{2}=-\frac{a_{3}}{4}-\frac{y_{6}}{2}+\frac{y_{8}}{2},\;\;\;\\ k_{3}=-\frac{a_{3}}{4}+\frac{y_{6}}{2}-\frac{y_{8}}{2},\;\;\;\;\;\;k_{4}=-\frac{a_{3}}{4}+\frac{y_{6}}{2}+\frac{y_{8}}{2},\; \end{array}$$

where

(57) $$\begin{array}{c} y_{0}=12a_{0}+a_{2}^{2}+3a_{1}a_{3},\;\;\;\;y_{1}=27a_{1}^{2}+72a_{0}a_{2}-2a_{2}^{3}-9a_{1}a_{2}a_{3}+27a_{0}a_{3}^{2},y_{2}=\frac{2a_{2}}{3}+\frac{a_{3}^{2}}{4},\\ \;\;y_{3}=8a_{1}-4a_{2}a_{3}-a_{3}^{3},\;\;\;\;y_{4}=\frac{\sqrt[{3}]{y_{1}+\sqrt{-4y_{0}^{3}+y_{1}^{2}}}}{3\sqrt[{3}]{2}},\;\;\;y_{8}=\sqrt{\frac{y_{7}}{4\sqrt{y_{6}}}}\\ y_{5}=\frac{\left(\frac{\sqrt[{3}]{2}y_{0}}{3}\right)}{\sqrt[{3}]{y_{1}+\sqrt{-4y_{0}^{3}+y_{1}^{2}}}},\;\;y_{7}=y_{2}-y_{5}-y_{4}-y_{3},\;\;y_{6}=\sqrt{y_{2}+y_{5}+y_{4}} \end{array}$$

The general solution of (53) is given by

(58) $$\bar{w}=\sum_{i=1}^{4}A_{i}e^{k_{i}z}+A_{5}e^{-\left(s/\upsilon \right)z}.$$

where $A_{i}$, i=1,2,3,4 are all parameters required to find out from the boundary conditions. Also, parameter $A_{5}$ is in the form

(59) $$A_{5}=\frac{\upsilon^{4}m_{0}}{s^{4}-a_{3}\upsilon s^{3}-a_{2}\upsilon^{2}s^{2}+\upsilon^{3}sa_{1}+\upsilon^{4}a_{0}},$$

Likewise, eliminating $\bar{w}$ between (48) and (49), we get

(60) $$\left(\frac{d^{4}}{dz^{4}}+a_{3}\frac{d^{3}}{dz^{3}}-a_{2}\frac{d^{2}}{dz^{2}}-a_{1}\frac{d}{dz}+a_{0}\right)\;\bar{\theta }=-m_{1}e^{-\left(s/\upsilon \right)z},$$

where

(61) $$m_{1}=h_{43}\left({\left(s/\upsilon \right)}^{2}-sN/\upsilon -h_{44}\right)$$

The general solution of Equation (50) is in the form

(62) $$\bar{\theta }=\sum_{i=1}^{4}A{{}^{\prime }}_{i}e^{k_{i}z}+A{{}^{\prime }}_{5}e^{-\left(s/\upsilon \right)z}.$$

Substitution of Equations (58) and (62) into Equation (47), we get the following relations

(63) $$A{{}^{\prime }}_{i}=\frac{h_{21}\left(k_{i}^{2}+Nk_{i}-h_{44}\right)}{h_{22}\left(k_{i}+N\right)}A_{i}=\Omega_{i}A_{i},$$

and

(64) $$A{{}^{\prime }}_{5}=\frac{h_{21}\left(s^{2}/\upsilon^{2}-Ns/\upsilon -h_{44}\right)}{h_{22}\left(-s/\upsilon +N\right)}A_{5}=\Omega_{5}A_{5}$$

At that moment, the thermodynamical temperature $\bar{\theta }$ in the Laplace domain with the help of the above equations, turn into

(65) $$\bar{\theta }=\sum_{i=1}^{4}\Omega_{i}A_{i}e^{k_{i}z}+\Omega_{5}A_{5}e^{-\left(s/\upsilon \right)z}.$$

After substituting Equations (58) and (62) into Equation (44), we obtain

(66) $$\frac{d\bar{\varphi }}{dz}=\sum_{i=1}^{4}F_{i}\;A_{i}e^{k_{i}z}+F_{5}\;e^{-\left(s/\upsilon \right)z}-\left(h_{13}/s\right)e^{-Nz},$$

where

(67) $$F_{i}=k_{i}+h_{12}\Omega_{i},\;\;i=1,2,3,4,\;\;\;F_{5}=\left(-s/\upsilon +h_{12}\Omega_{5}\right)A_{5}.$$

Thus the solution for electric potential $\bar{\varphi }$ in Laplace transform, the domain can be obtained

(68) $$\bar{\varphi }=\sum_{i=1}^{4}F_{i}\;A_{i}\;e^{k_{i}z}/k_{i}-\left(\upsilon F_{5}/s\right)\;e^{-\left(s/\upsilon \right)z}+\left(h_{13}\;/Ns\right)e^{-Nz}+A_{0}$$

Subsequently, after getting the final solutions for electric potential $\bar{\varphi }$, displacement $\bar{w}$ and temperature $\bar{\theta }$, the normalized stress ${\bar{\sigma }}_{zz}$ and electric displacement $\bar{E}$ in the Laplace domain may be achieved using Equations (10) and (38) as:

(69) $${\bar{\sigma }}_{zz}=e^{Nz}\left[\sum_{i=1}^{4}\left(h_{21}k_{i}-h_{22}\Omega_{i}\right)A_{i}e^{k_{i}z}-\left(h_{21}s/\upsilon +h_{22}\Omega_{5}\right)A_{5}e^{-\left(s/\upsilon \right)z}\right]-h_{23}/s,.\;$$

(70) $$\bar{E}=-\sum_{i=1}^{4}F_{i}\;A_{i}e^{k_{i}z}-F_{5}e^{-\left(s/\upsilon \right)z}+\left(h_{13}/s\right)e^{-Nz},$$

Introducing the boundary conditions (42) and (43) in (58), (62) and (68) after using Laplace transform, we obtain six equations in the unknown parameters $A_{i}$, i=1,2,3.4 and $A_{0}$ as

(71) $$A_{1}+A_{2}+A_{3}+A_{4}=-A_{5},$$

(72) $$A_{1}e^{k_{1}L}+A_{2}e^{k_{2}L}+A_{3}e^{k_{3}L}+A_{4}e^{k_{4}L}=-A_{5}e^{-\left(s/\upsilon \right)L},$$

(73) $$A_{1}\Omega_{1}+A_{2}\Omega_{2}+A_{3}\Omega_{3}+A_{4}\Omega_{4}=-B_{5}$$

(74) $$A_{1}\Omega_{1}e^{k_{1}z}+A_{2}\Omega_{2}e^{k_{2}z}+A_{3}\Omega_{3}e^{k_{3}z}+A_{4}\Omega_{4}e^{k_{4}z}=-B_{5}e^{-\left(s/\upsilon \right)L}.$$

(75) $$\frac{F_{1}}{k_{1}}A_{1}+\frac{F_{2}}{k_{2}}A_{2}+\frac{F_{3}}{k_{3}}A_{3}+\frac{F_{4}}{k_{4}}A_{4}-\left(\frac{\upsilon F_{5}}{s}\;-\frac{h_{13}}{Ns}\right)+A_{0}=0$$

After investigating the solutions of the above equations, we have the values of the unknown parameters $A_{i}$, whose solution solves the problem in the Laplace transform field.

## 6. Homogenous Case

The solution to the homogeneous materials can be obtained by neglecting the nonhomogeneity parameter (N=0). In this case, the differential Equations (53), (60) and (66) will be in the following forms

(76) $$\left(\frac{d^{4}}{dz^{4}}-b_{2}\frac{d^{2}}{dz^{2}}+b_{0}\right)\;\bar{w}=f_{0}e^{-\left(s/\upsilon \right)z},$$

(77) $$\left(\frac{d^{4}}{dz^{4}}-b_{2}\frac{d^{2}}{dz^{2}}+b_{0}\right)\;\bar{\theta }=-f_{1}e^{-\left(s/\upsilon \right)z},$$

(78) $$\frac{d\bar{\varphi }}{dz}=\sum_{i=1}^{4}F_{i}\;A_{i}e^{k_{i}z}+F_{5}\;e^{-\left(s/\upsilon \right)z}-\left(h_{13}/s\right),$$

Then general solutions of all studied fields will be in the forms

(79) $$\bar{w}=\sum_{i=1}^{4}B_{i}e^{q_{i}z}+B_{5}e^{-\left(s/\upsilon \right)z}.$$

(80) $$\bar{\theta }=\sum_{i=1}^{4}\psi_{i}B_{i}e^{q_{i}z}+\psi_{5}B_{5}e^{-\left(s/\upsilon \right)z}.$$

(81) $$\bar{\varphi }=\sum_{i=1}^{4}g_{i}\;B_{i}\;e^{q_{i}z}/q_{i}-\left(\upsilon g_{5}/s\right)\;e^{-\left(s/\upsilon \right)z}-\left(h_{13}\;/s\right)z+B_{0}$$

(82) $${\bar{\sigma }}_{zz}=\sum_{i=1}^{4}\left(h_{21}k_{i}-h_{22}\psi_{i}\right)B_{i}e^{q_{i}z}-\left(h_{21}s/\upsilon +h_{22}\psi_{5}\right)B_{5}e^{-\left(s/\upsilon \right)z}-h_{23}/s,$$

(83) $$\bar{E}=-\sum_{i=1}^{4}g_{i}\;B_{i}e^{k_{i}z}+g_{5}\;e^{-\left(\frac{s}{\upsilon }\right)z}+\left(h_{13}/s\right),$$

where

(84) $$\begin{array}{c} \;b_{2}=h_{41}+h_{44}+\frac{h_{42}h_{22}}{h_{21}},\;\;a_{0}=h_{44}h_{41},\;\;\;f_{0}=\frac{sh_{43}h_{22}}{\left(h_{21}v\right)},\\ \;f_{1}=h_{43}\left({\left(s/\upsilon \right)}^{2}-h_{44}\right),\;\;\;\psi_{i}=\frac{h_{21}\left(k_{i}^{2}-h_{44}\right)}{k_{i}h_{22}},\;\;\psi_{5}=\;-\frac{\upsilon h_{21}\left(s^{2}/\upsilon^{2}-h_{44}\right)}{sh_{22}}\;,\\ q_{i}=\pm \sqrt{\frac{1}{2}\left(b_{2}\pm \sqrt{b_{2}^{2}-4b_{0}}\right)},B_{5}=\frac{\upsilon^{4}f_{0}}{s^{4}-a_{3}\upsilon s^{3}-a_{2}\upsilon^{2}s^{2}+\upsilon^{3}sa_{1}+\upsilon^{4}a_{0}},\\ g_{i}=k_{i}+h_{12}\Omega_{i},\;\;\;\;g_{5}=\left(-\frac{s}{\upsilon }+h_{12}\Omega_{5}\right)A_{5},\;\;\;i=1,2,3,4. \end{array}$$

Introducing boundary conditions (42) and (43) in (58), (62) and (68), we obtain the values of the unknown parameters $B_{i}$, i=1,2,3.4 and $B_{0}$.

## 7. Numerical Inversion of the Laplace-Transformed Equations

In the physical domain, to get the solution of the current considered problem, the transforms of the governing equations need to be inverted. We adopt a numerical inversion method based on a Fourier series expansion [49] for the inversion of the Laplace transform in the above equations. In this procedure, any function in the Laplace domain may be reversed to the considered time domain as

(85) $$\psi \left(t\right)=\frac{e^{ct}}{t}\left(Re\left[\frac{\bar{\psi }\left(c\right)}{2}\right]+Re\left[\sum_{n=1}^{n_{0}}\bar{\psi }\left(c+in\pi /t\right){\left(-1\right)}^{n}\right]\right),$$

where Re and i, respectively are the real part and imaginary number unit and $n_{0}$ is a finite number. To ensure the fast convergence, many numerical experiments have been given away that the value of ***C*** satisfies the relation ct≅4.7 [50].

## 8. Numerical Analysis and Discussion

In order to clarify the analytical procedure previously presented and to compare the theoretical results attained in the earlier sections, a numerical example is considered and the computational results are presented. The chosen material for this numerical assessment is cadmium selenide, which is known as a graded material. Thus the physical properties of the problem have been specified in SI units [51] as

$$\begin{array}{c} C_{0}=7.41\times \;10^{10}\;{N/m}^{2},\beta_{0}=0.621\times \;10^{6}\;{N/\mathrm{Km}}^{2},e_{0}=0.347\;{C/m}^{2},\;T_{0}=293\;K,\;\\ p_{0}=-2.94\times \;10^{-6}\;{C/\mathrm{Km}}^{2},\;\;\;\rho_{0}=7600\;\mathrm{kg}\;m^{-3},\;Q_{0}=10/\rho_{0},K_{0}=12.9\;W/\mathrm{mK},\\ C_{E}=420\;J/\mathrm{kgK},\;\;\in_{0}=90.3\times \;10^{-12}\;C^{2}/\mathrm{Nm},\;\;\;L=1. \end{array}$$

The numerical method described above was used to get the displacement w, electric potential φ, temperature θ and the normalized stress $\sigma_{zz}$ distributions inside the medium. The obtained numerical results are demonstrated graphically in Figure 1, Figure 2, Figure 3, Figure 4, Figure 5, Figure 6, Figure 7, Figure 8, Figure 9, Figure 10, Figure 11, Figure 12, Figure 13, Figure 14, Figure 15, Figure 16, Figure 17, Figure 18, Figure 19 and Figure 20. From the drawn Figures, we noticed that all field quantities rely not only on the state and variables t and z but also depend on the velocity of the heat source υ, the thermal relaxation time $\tau_{0}$ and nonhomogeneity parameters N. The mathematical calculations are performed out for the three different cases, described below:

### 8.1. Effects of Memory-Dependent Factors

**Figure 1:**
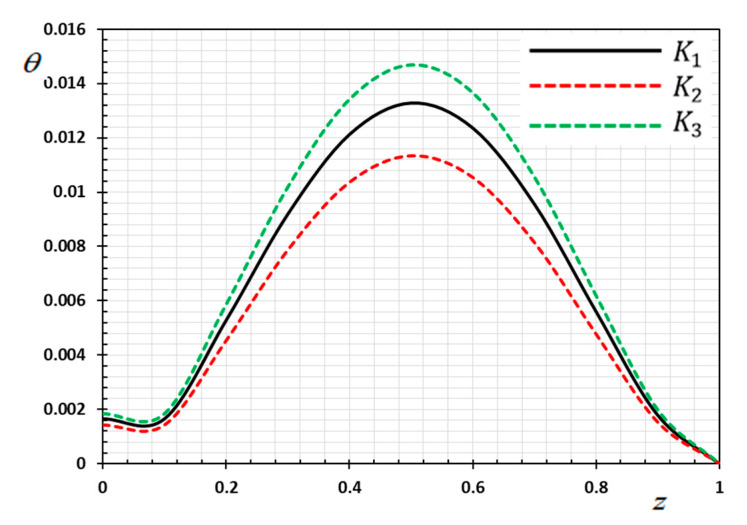
Variation of temperature θ versus z for different forms of kernel function k(t−ξ).

**Figure 2:**
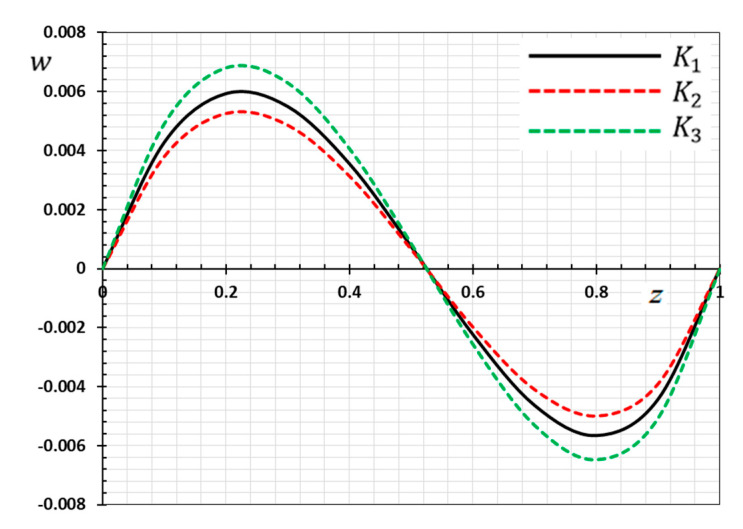
Variation of displacement w versus z for different forms of kernel function k(t−ξ).

**Figure 3:**
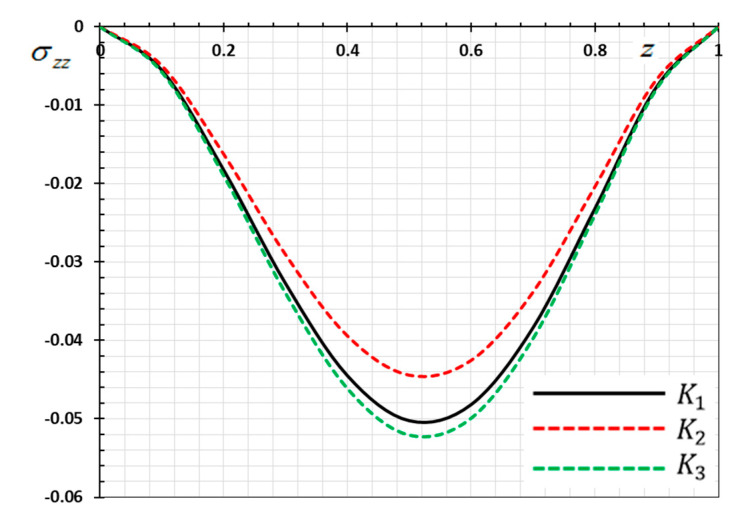
Variation of thermal stress $\sigma_{zz}$ versus z for different forms of kernel function k(t−ξ).

**Figure 4:**
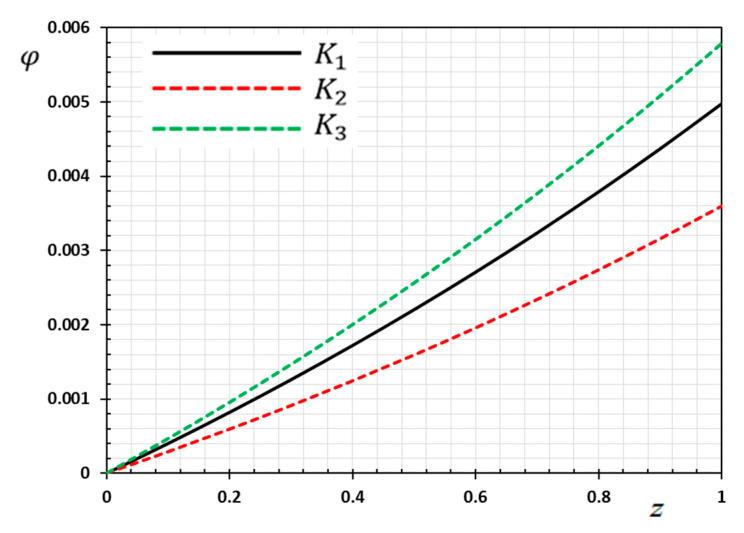
Variation of electric potential φ versus z for different forms of kernel function k(t−ξ).

**Figure 5:**
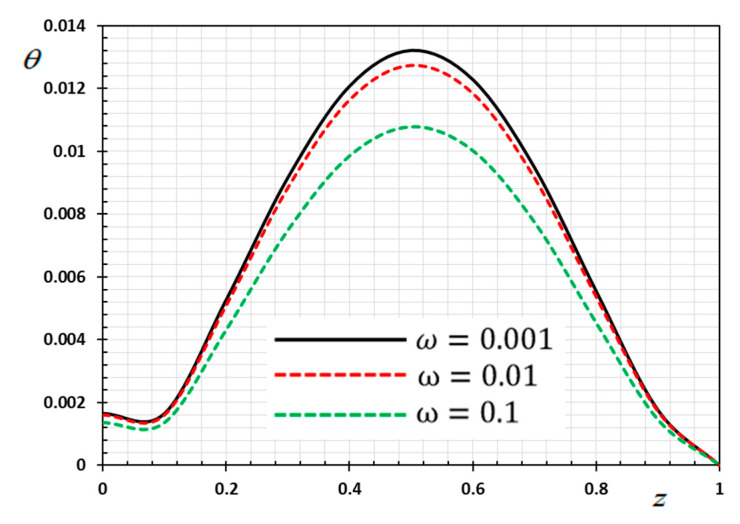
Variation of temperature θ versus z for different delay time ω.

**Figure 6:**
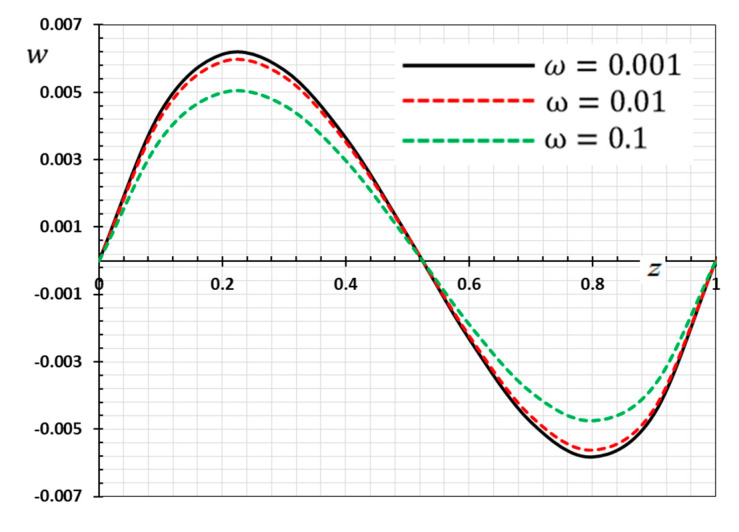
Variation of displacement w versus z for different delay time ω.

**Figure 7:**
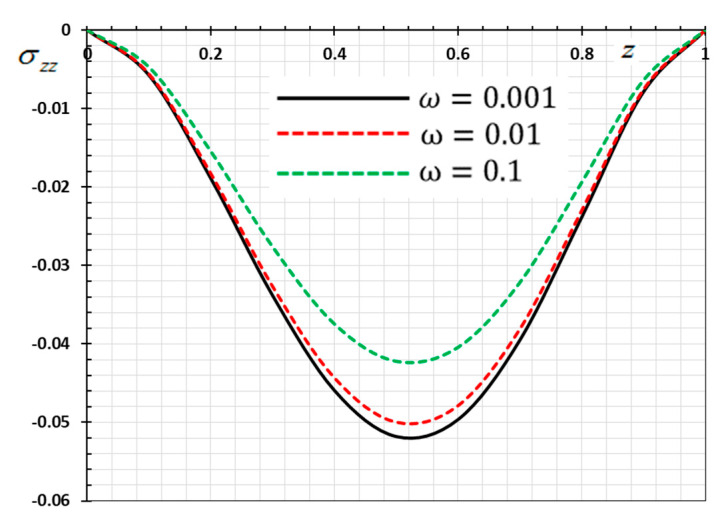
Variation of thermal stress $\sigma_{zz}$ versus z for different delay time ω.

**Figure 8:**
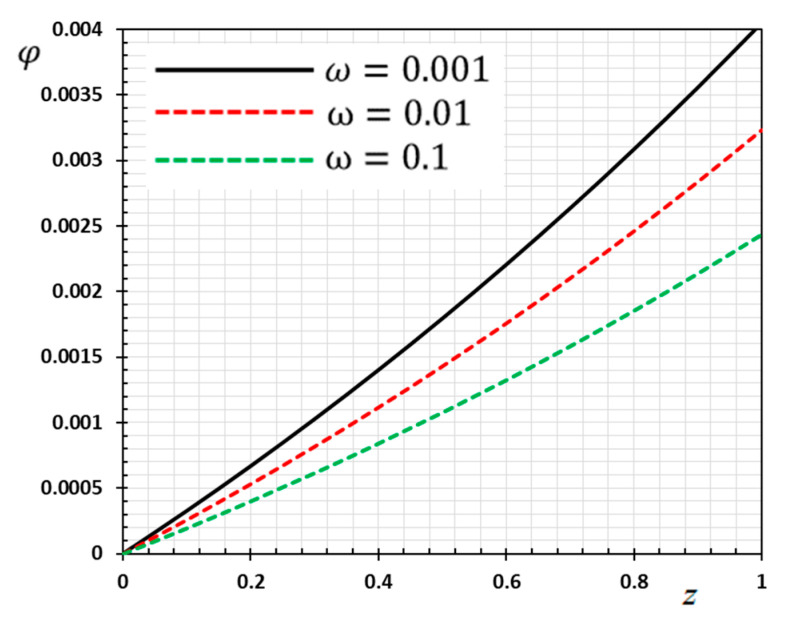
Variation of electric potential φ versus z for different delay time ω.

The effects of memory-dependent factors (kernel function k(t−ξ) and time-delay parameter ω) on all field quantities (φ, θ, w and $\sigma_{zz}$) versus the distance ξ are discussed in this case. For this purpose of the study, two sets of figures have been presented. The first group of Figure 1, Figure 2, Figure 3 and Figure 4 illustrates how the behavior of all physical thermal fields changes, taking into account different forms of the kernel function. The second group attempted to illustrate the effect of the time delay on the response of all physical fields through Figure 5, Figure 6, Figure 7 and Figure 8. The calculations are carried out for υ=0.01, ω=0.01, $\tau_{0}=0.1$ and N=0.3.

By the definition of the MDD and the numerical results that we obtained, we noticed that the kernel function is one of the significant aspects that affect the solutions and behavior of the studied fields. The kernel function is selected as $k\left(t-\xi \right)=1-\frac{2b}{\omega }\left(t-\xi \right)+\frac{a^{2}{\left(t-\xi \right)}^{2}}{\omega^{2}}$. The comparison will mainly be made on the influence of the kernel function on the nature of the performance of the fields when the kernel function is linear (a=0) and quadratic (a>0) functions. The calculations are done for the three kernel functions as $K_{1}=1$, $K_{2}=1-\frac{1}{\omega }\left(t-\xi \right)$ and $K_{3}={\left(1-\frac{t-\xi }{\omega }\right)}^{2}$.

Figure 1 shows the temperature variance θ versus the distance concerning the various selections of the kernel functions under the influences of the moving heat supply. From Figure 1, we note that the temperature distribution increases with increasing time and distance until it reaches its maximum value, then decreases again gradually, approaching zero. The thermal distribution is very similar to the normal distribution and may be the cause of these moving heat sources. Similarly, it is noticed that the magnitude of temperature θ decomposes faster for the kernel function $K_{2}$ compared to the kernel $K_{1}$ than that of the values of $K_{3}$.

Figure 2 is depicted to display the difference in the distance z versus the displacement values w for the same selections of kernel functions k(t−ξ), as stated previously. We noticed that the displacement distribution is an oscillating distribution that is very similar to the sine function. The displacement variation was detected to increase in 0<z<0.22 and then to decompose away from the plane z=0.22 to reach the minimum value near z=0.8, and finally it increased again in 0.8<z<1.0. It is observed from the figure that the displacement vanishes on the surfaces z=0, L, which satisfies the proposed mechanical boundary conditions, as shown in Equation (44). It can be found from Figure 2 that the peak values of the magnitude of w are greater for $K_{3}$ than $K_{2}$, compared the peak of $K_{2}$.

Figure 3 displays the nondimensional stress distribution $\sigma_{zz}$ in the thermo-piezoelectric rod for different kernel functions. As investigated in Figure 3, the stress distribution is compressive within the medium due to the fixed ends of the rod. It is also observed that the absolute value of thermal stress increases with time till it reaches the extreme value, and at that time gradually decreases again, completely reversing the behavior of temperature. Note that the pressure size in the case of the nonlinear kernel $K_{3}$ is the smallest compared with the other kernel $K_{1}$ than $K_{2}$.

Figure 4 is depicted to show the variant of the electric potential φ against z for different forms of kernel functions k(t−ξ). From Figure 4, it is observed that the electric potential values increase with increasing z. In the figure, all curves of φ begin with zero values and meet the proposed boundary condition that φ=0 at z=0. Without the memory effect, it was observed that the electric potential φ was slower compared to the memory effect. Moreover, it is noted that the occurrence of the heat source Q affects the electric potential increase. Also, the magnitude of the displacement profile is greater for the kernel $K_{3}$, than that of the kernels $K_{1}$ and $K_{2}$.

It has been found that the nature of distributions in all physical field in the current study and the corresponding outcomes of generalized thermoelasticity with a single memory-dependent derivative relaxation parameter is consistent with the existence of the physical field variable distribution for both Lord and Shulman as derived from Ezzat and El-Bary [41] and Mondal and Kanoria [52].

In contrast to other thermoelastic models including fractional-order or integral order derivative, the use of Integrated MDD transforms provides more accurate, detailed and continuous numerical findings.

Figure 5, Figure 6, Figure 7 and Figure 8 are drawn to display the influence of the delay time ω on the studied fields for the kernel function $K_{3}={\left(1-\frac{t-\xi }{\omega }\right)}^{2}$ because of the moving heat source. For illustration purposes, the other specific parameters are considered to be υ=0.01, $\tau_{0}=0.1$ and N=0.3 and the delay time take three different values ω=0.001, 0.01 and 0.1, respectively. The delay time ω plays a significant role in the propagation of the considered fields. As shown from the figures, the increase in the delay time value increases the magnitudes of the physical variable profiles inside the thermo-piezoelectric rod.

### 8.2. Nonhomogeneous Parameter Effect

**Figure 9:**
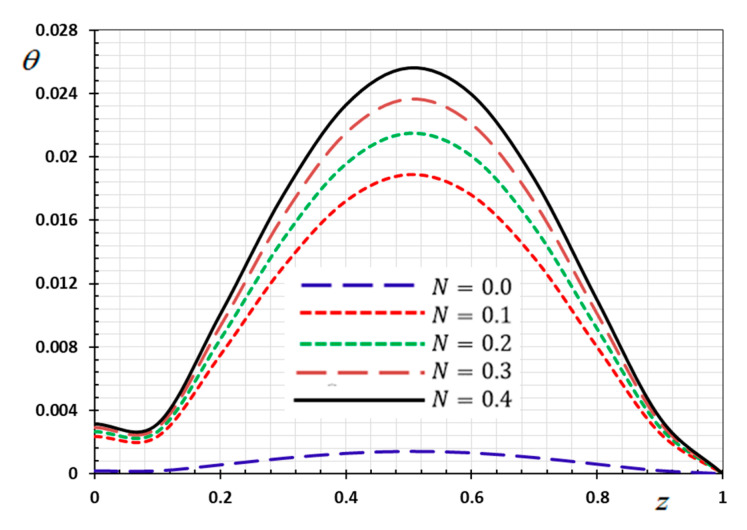
Variation of temperature θ versus z for different gradient coefficient N.

**Figure 10:**
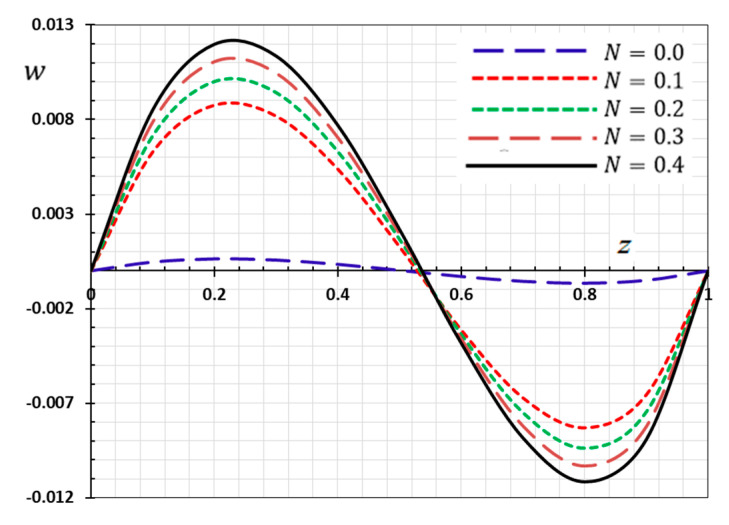
Variation of displacement w versus z for different gradient coefficient N.

**Figure 11:**
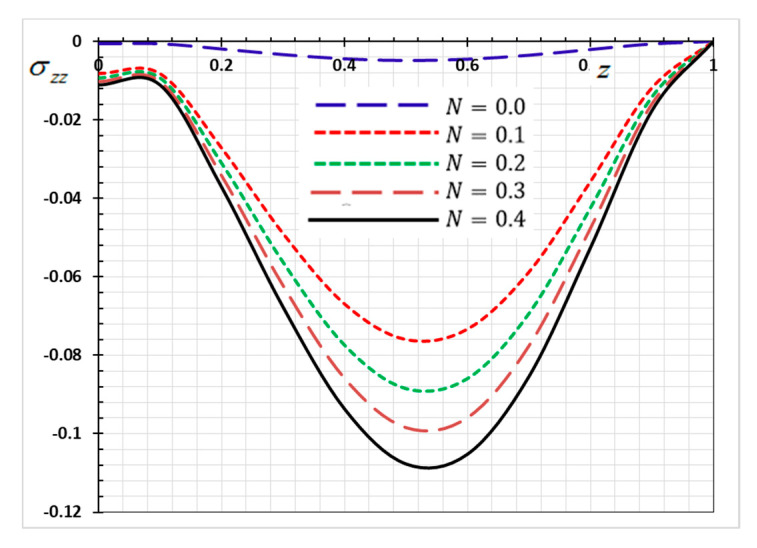
Variation of Thermal stress $\sigma_{zz}$ versus z for different gradient coefficient N.

**Figure 12:**
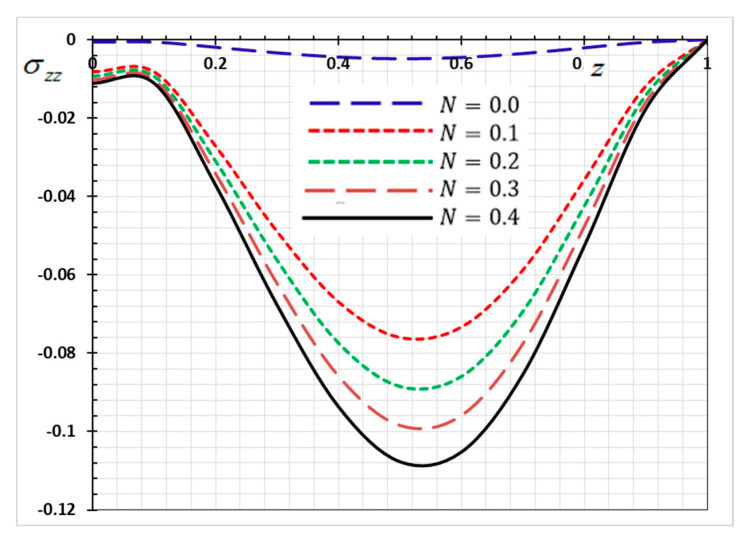
Variation of electric potential φ versus z for different gradient coefficient N.

To discuss the influence of nonhomogeneous parameter N on the thermophysical quantities, Figure 9, Figure 10, Figure 11 and Figure 12 are presented. The numerical results are displayed when the effective parameters υ=0.05, $\tau_{0}=0.1$ and ω=0.01 are fixed, as the kernel function is nonlinear $K_{3}={\left(1-\frac{t-\xi }{\omega }\right)}^{2}$. We note that when the nonhomogeneous parameter is set to zero (N=0), we get the old state in which the body is homogeneous, has normal properties and is not graded functionally. In this case, we consider four different values for the nonhomogeneous index N in addition to the normal case (N=0). It is noted from the Figures that the parameter of the gradient index N affects the displacement, electric potential, temperature and the normalized stress variations in the functionally graded rod.

Figure 9 displays the relation between the temperature values θ and the index of the material proprieties N along the axial axis z. Figure 6 shows that the temperature change θ increases with increasing heterogeneous parameter values 9. From the important observations that we obtained about this Figure, it is clear that the behavior of heat transfer within homogeneous materials differs significantly from that in functionally graded piezoelectric materials (FGPM).

This observation indicates that an ideal FGPM rod can be planed by choosing an appropriate material gradient index N which is the advantage of the FGPM structure.

In Figure 10, the displacement w of the FGM rod are presented with different nonhomogeneous parameter N. It is noted further that the displacement absolute w increases when the nonhomogeneity parameter N increases. Due to the fixed ends of the rod, it is noted that the deformation is restricted between both ends, resulting in compressed thermal stress in the rod.

The numerical results of the nondimensional normalized stress $\sigma_{zz}$ of the functionally graded rod along the z-axis for various values of the nonhomogeneity parameter N are revealed in Figure 11. As shown in Figure 11, a significant difference in the stress was observed with the change in the values of the nonhomogeneity parameter N. From Figure 3, it may be found that the absolute of the stress $\sigma_{\mathrm{ZZ}}$ increases with the increase of the parameter N.

The above results are of great importance in thermal engineering applications, such as safety design of the electronic or mechanical devices under severe thermal loadings [53].

Figure 12 is plotted to show the electric potential φ variation of the functionally graded rod compared to z for different values of gradient indicator N. From the curves in Figure 12, it is verified that the values of φ increase with increasing z. Moreover, it was noticed that the presence of the gradient coefficient N affects the electric potential increase.

One of the goals for introducing functionally graded materials is to reduce thermal stresses in such structures operating in a high-temperature environment. Consequently, investigating the effects of the nonhomogeneity parameter on thermophysical quantities is important for designing FG materials.

In comparison with the previous literature, it was found that the nature of all the distributions of physical fields in the current study and the corresponding results of generalized thermal elasticity with one derivative relaxation coefficient without relying on memory is consistent with the numerical results and the nature of the distributions as stated in [54].

### 8.3. The Influence of the Velocity of the Heat Source

**Figure 13:**
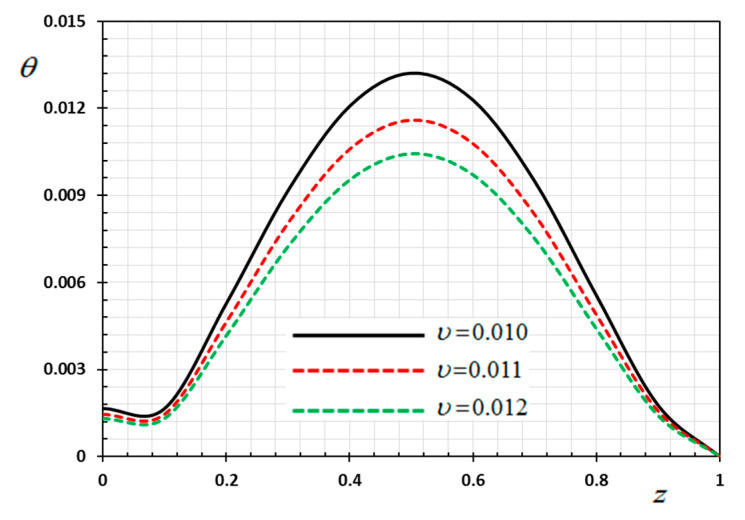
Variation of temperature θ versus z for different velocity of the heat source υ.

**Figure 14:**
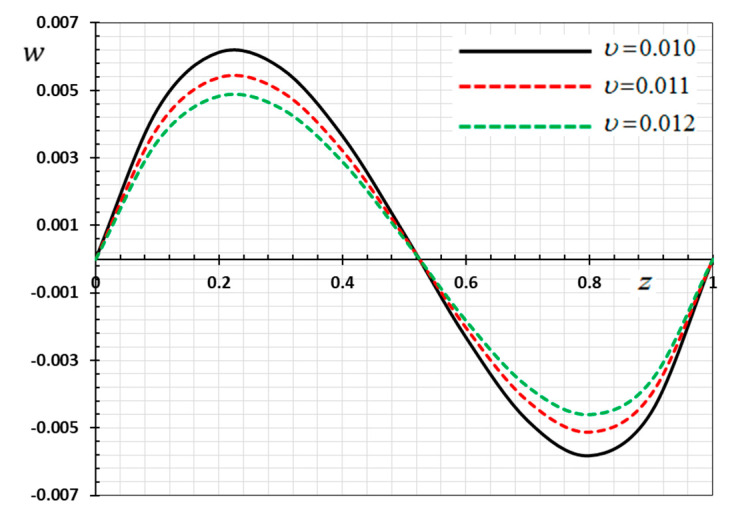
Variation of displacement w versus z for different gradient coefficient N.

**Figure 15:**
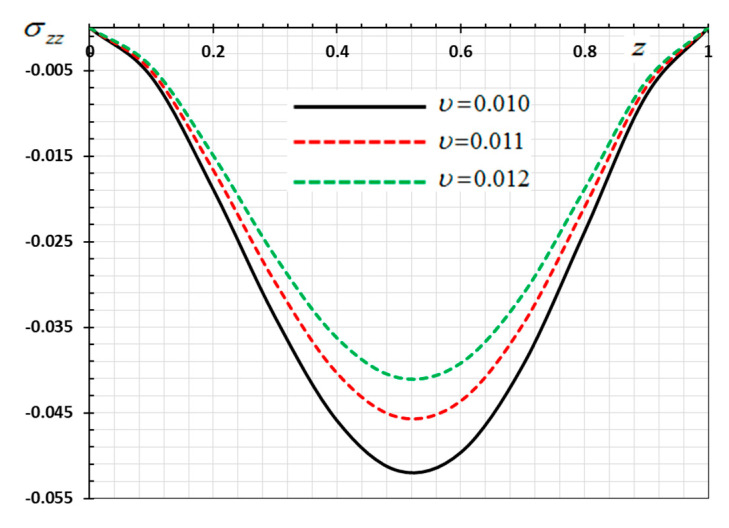
Variation of thermal stress $\sigma_{zz}$ versus z for different velocity of the heat source υ.

**Figure 16:**
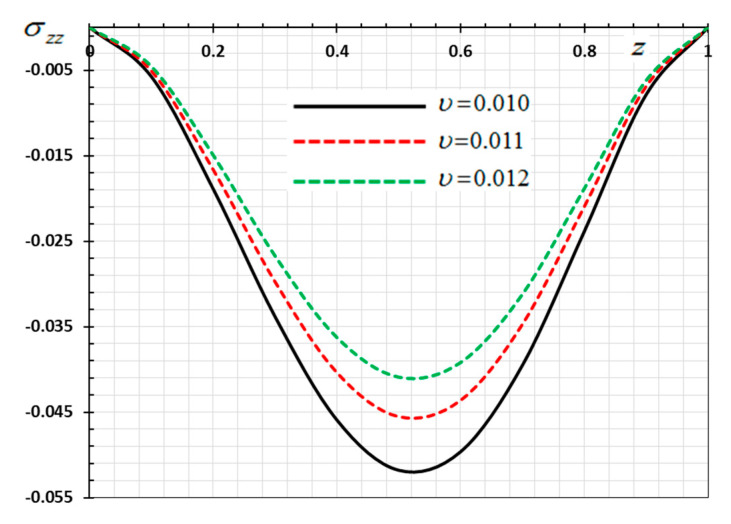
Variation of electric potential φ versus z for different velocity of the heat source υ.

In this case, the influence of the velocity of movable heat source υ on the distribution of the field variables (φ, θ, w and $\sigma_{zz}$) have been studied. The variations for the different distributions are shown in Figure 13, Figure 14, Figure 15 and Figure 16. The numerical results are calculated when the other parameters are fixed (N=0.3, $\tau_{0}=0.1$ ω=0.01) and in the case of $K_{3}={\left(1-\frac{t-\xi }{\omega }\right)}^{2}$. As an example of the effect of the parameter υ, three different values of the parameter υ are taken into consideration, which are υ=0.1, υ=0.11, and υ=0.012. From Equation (35) it is clear that the heat source emits its maximum energy at the position z=tυ, which result in the peak value. We detected that the various values of the parameter of the heat supply speed υ have an important effect on all fields. It has been observed that the different values of the heat source velocity parameter v (2.0, 3.0, 4.0) have an important impact on all physical fields. As shown in Figure 13, Figure 14, Figure 15 and Figure 16, the magnitudes the nondimensional variables increase with increasing velocity of the moving heat resource. The effects of the heat source transmission speed υ on all the considered quantities are very large, as obviously shown by the greatest values of the curves. The effect of the heat source is an intrinsic factor that cannot be ignored when assessing heat stress and fields at the source of moving heating problems [54].

### 8.4. The Effect of the Thermal Relaxation Time

**Figure 17:**
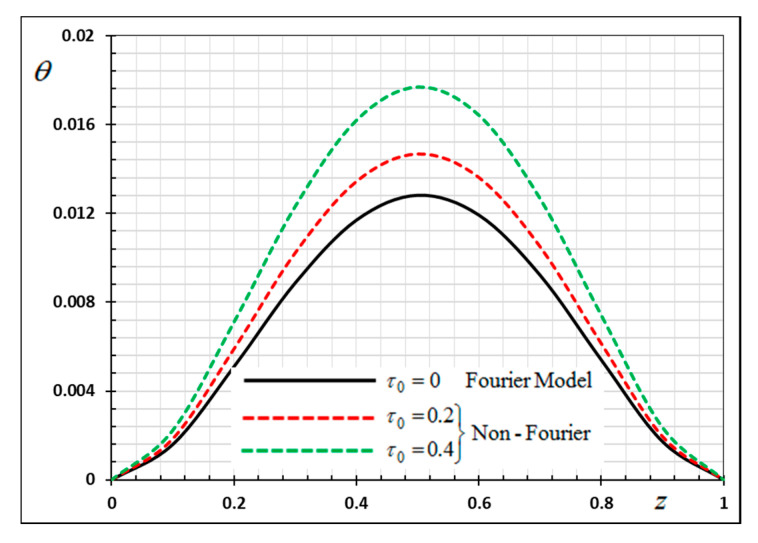
Variation of temperature *θ* versus *z* for Fourier and non-Fourier models.

**Figure 18:**
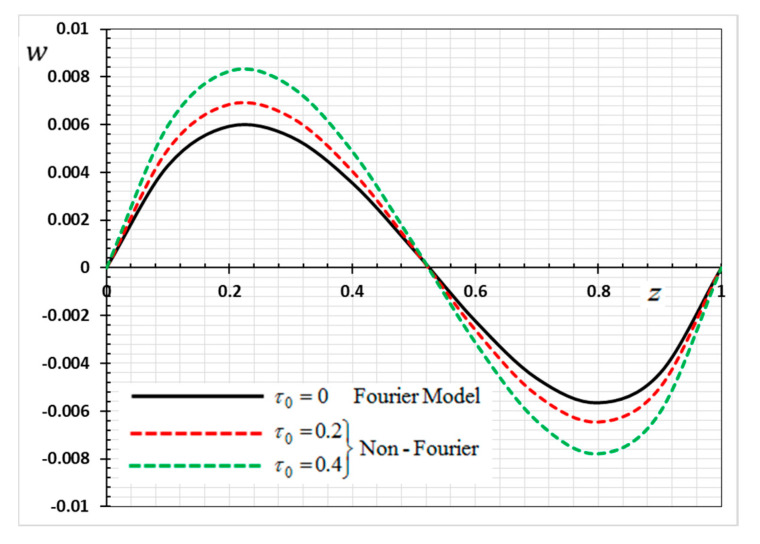
Variation of displacement *w* versus *z* for Fourier and non-Fourier models.

**Figure 19:**
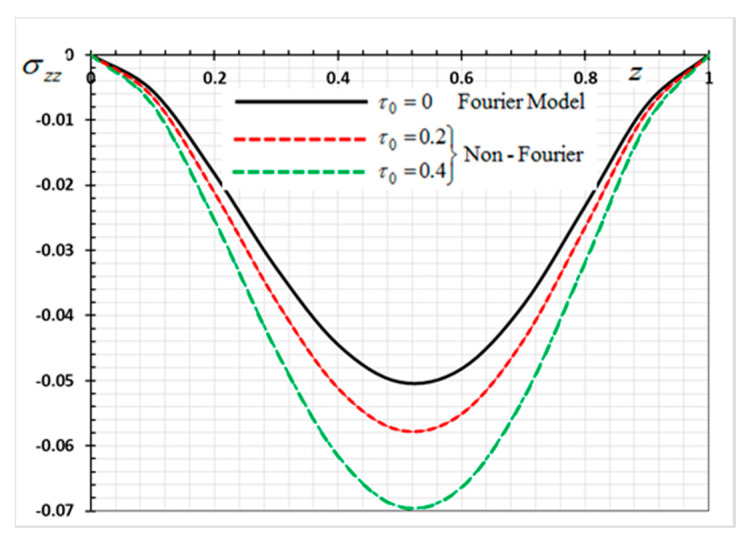
Variation of thermal stress *σ_zz* versus *z* for Fourier and non-Fourier models.

**Figure 20:**
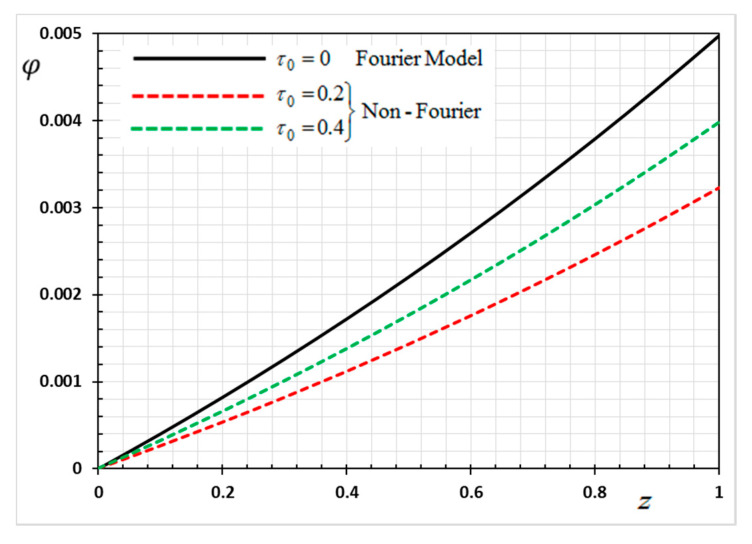
Variation of electric potential *φ* versus *z* for Fourier and non-Fourier models.

In the last case, we will make a comparison of the different physical fields within the functionally graded piezoelectric materials in the case of the classic Fourier Law (CTE) and the generalized theory of thermoelasticity (hyperbolic non-Fourier Law) proposed by Lord and Shulman (LS). The coupled theory of thermoelasticity (CTE) can be obtained when there no rate of heat flux appear (in Equation (5) $\tau_{0}=0$ and we can obtain the generalized model (LS) if $\tau_{0}>0$. When single-phase delay is absent ($\tau_{0}=0$), this also leads to the absence of a memory effect ($D_{\omega }=0)$. Other effective parameters are assumed to be fixed during the numerical calculation. The comparisons are represented by Figure 17, Figure 18, Figure 19 and Figure 20.

It can be seen that the single-phase delay parameter $\tau_{0}$ has a significant effect on the distribution of all studied fields. The mechanical behaviour of the studied fields shows that the wave propagates with the limited speed in the medium.

The values differ in the classic theory of thermoelasticity (CTE) compared to the values of the other model (LS). The magnitudes of variables studied in the LS model are larger compared to the CTE model. The fact that in generalized thermoelasticity model (LS), the thermal and mechanical waves propagate at limited velocities are obvious in all of these figures. The behavior of the two models is generally similar.

## 9. Conclusions

In this paper, the dynamic piezoelectric responses of a thermoelastic functionally graded rod exposed to a movable heat source are studied based on the generalized heat conduction with a memory-dependent derivative involving time-delay and a kernel function. The physical properties of the graded piezoelectric rod vary according to exponential functions in the axial direction of the rod. Using Laplace transform as well as its inversion techniques, solutions to the physical variables have been obtained numerically.

The results are validated compared to previous studies. Numerical results show significant effects of the speed of the heat source, the power index, the kernel function and the time-delay parameter on the distribution of the studied functions. For assessment, the results of the classical Fourier heat conduction without memory influence are obtained as well.

From the obtained results of this analysis and according to the introduced model, we can classify the materials, whether homogeneous or functionally graded in terms of propagation of mechanical and thermal waves depending on to delay of time as well as the various forms of the function of the kernel.

In addition, the new MDD in this model can perform an important role in studying the behavior of some materials that predominate in determining the physical properties of the materials.

Finally, the nonhomogeneity parameter offered in the present study is a useful parameter from the design point of view in that it can be designed for specific applications to control the distributions of temperature and thermoelastic stresses.
